# Soluble N-Acetylgalactosamine-Modified Antigens Enhance Hepatocyte-Dependent Antigen Cross-Presentation and Result in Antigen-Specific CD8^+^ T Cell Tolerance Development

**DOI:** 10.3389/fimmu.2021.555095

**Published:** 2021-03-03

**Authors:** Martina Damo, D. Scott Wilson, Elyse A. Watkins, Jeffrey A. Hubbell

**Affiliations:** ^1^Institute for Molecular Engineering, University of Chicago, Chicago, IL, United States; ^2^Institute for Bioengineering, School of Life Sciences and School of Engineering, Ecole Polytechnique Fédérale de Lausanne (EPFL), Lausanne, Switzerland

**Keywords:** hepatocytes, tolerance, cross-presentation/priming, antigen modifications, CD8 T cell

## Abstract

Hepatocytes compose up to 80% of the total liver and have been indicated as important players in the induction of immunologic tolerance in this organ. We show that hepatocytes possess the molecular machinery required for the cross-presentation of extracellular antigens. Using a derivative of the model antigen ovalbumin (OVA) covalently modified with a polymer containing multiple N-acetylgalactosamine residues (pGal-OVA) that enhance extracellular antigen uptake by mimicking the glycome of apoptotic debris, we show efficient hepatocyte-dependent induction of cross-tolerance of both adoptively transferred OT-I cells and endogenous OVA-specific CD8^+^ T lymphocytes, for example inducing tolerance to OVA-expressing skin transplants. Our study confirms that hepatocytes are capable of inducing peripheral tolerogenesis and provides proof of concept that they may be a valuable candidate for *in vivo* targeted tolerogenic treatments.

**Significance Statement**

Liver has been long known for its role in maintaining immunological tolerance toward circulating antigens, however this function has been mainly attributed to liver endothelial cells. Our study highlights that primary hepatocytes express the molecular machinery for up-taking, processing, and presenting extracellular antigens on the MHC-I, thus leading to cross-presentation of extracellular antigens. Through antigen cross-presentation, hepatocytes can thus induce tolerance of antigen-specific CD8^+^ T cells *in vivo*. In a model of skin transplantation, hepatocyte-dependent antigen cross-presentation is also capable of delaying or completely preventing the rejection of skin grafts expressing the cognate antigen. Thus, our data suggest that hepatocytes can be valuable candidates for the development of effective treatments aimed at inducing immunological tolerance.

## Introduction

The liver is known to be involved in a variety of tolerogenic processes, for example to harmless non-self antigens absorbed into the blood draining from the gut or newly formed antigens resulting from hepatic metabolic activities, which fail to induce an immune response in healthy individuals (1, 2). Antigen-specific tolerance and cross-tolerance induction toward CD4^+^ and CD8^+^ T cells, respectively, has been attributed to liver sinusoidal endothelial cells (LSECs), which as MHC-I- and MHC-II-expressing blood vessel-lining cells represent the first cells to interact with peripheral lymphocytes entering the hepatic circulation. LSECs efficiently scavenge, process and present soluble antigens found in the bloodstream to circulating lymphocytes, typically resulting in the induction of CD4^+^ regulatory T cells or anergic CD8^+^ T cells (3–8).

Unlike other organs, where circulating lymphocytes only extravasate and gain access to the parenchyma in the case of inflammation, the liver microvasculature has a peculiar fenestrated endothelium devoid of any basal membrane, allowing direct physical contact between circulating CD8^+^ T lymphocytes and liver MHC-I^+^ parenchymal cells, the hepatocytes (9). It has been reported that hepatocytes possess poor cross-presentation capacity *in vitro* as compared to other liver cells, especially LSECs (6). Nonetheless, several publications have shown that direct antigen expression, obtained by either transgenesis or viral vector transduction, and subsequent MHC-I-dependent antigen presentation in hepatocytes *in vitro* and *in vivo* can result in immune tolerance mainly by suboptimal activation of antigen-specific CD8^+^ T lymphocytes because of the lack of CD28 co-stimulation leading to clonal deletion of the T cells (10–16). Moreover, other reports also described the induction of FoxP3^+^ Treg cells upon recombinant viral vector-mediated hepatocyte-dependent antigen presentation, thus indicating a possible involvement of other antigen-presenting cells (APCs) in hepatocyte-driven tolerogenic mechanisms, since hepatocytes lack MHC-II expression to interact with CD4^+^ T cells directly (16–18).

As hepatocytes outnumber the other cellular components of the liver and are in close contact with components of the blood, hepatocytes have been proposed to participate in the establishment of CD8^+^ T cell peripheral tolerance through mechanisms of extracellular antigen uptake and cross-presentation. For example, hepatocytes possess lectin receptors, including the asiaoglycoprotein receptor (ASGRP) (19). Apoptotic processes activate neuraminidases that desialylate glycoproteins to expose terminal N-acetylgalactosamine residues, which bind to ASGPR (19–22); given the peripheral tolerogenic nature of apoptotic debris (23, 24), this further motivated our interest in evaluating how to exploit hepatocytes to efficiently process and tolerogenically present circulating exogenous antigens (in our model, N-acetylgalactosaminylated antigens, 25). In this study, we take advantage of a chemically engineered form of Ovalbumin (OVA) to enhance both *in vitro* and *in vivo* the cross-presentation capabilities of murine hepatocytes, thus describing the immunological consequences of hepatocyte-dependent antigen cross-presentation *in vivo*. Our results demonstrate efficient hepatocyte-dependent induction of CD8^+^ T cell tolerance toward an extracellular antigen and suggest the relevance of hepatocytes as interesting target cells for tolerogenic prophylactic or therapeutic interventions.

## Results

### Primary Hepatocytes Efficiently Use EEA1- and TAP1-Positive Cytoplasmic Compartments for Processing Extracellular Antigens

Cross-presentation is the result of antigen uptake mediated by Fc or C-type lectin receptors (such as the mannose receptor), followed by antigen proteolytic degradation by proteasomes associated with early endosome antigen 1 (EEA1)-positive phagosomes (early endosomes), subsequent antigen transport and loading onto MHC-I molecules through transporters associated with antigen processing (TAP) and translocation of peptide/MHC-I complexes to the cell plasma membrane via the secretory pathway (25–27). We asked whether hepatocytes would employ cross-presentation-competent subcellular compartments to process soluble extracellular antigens.

**Figure 1:**
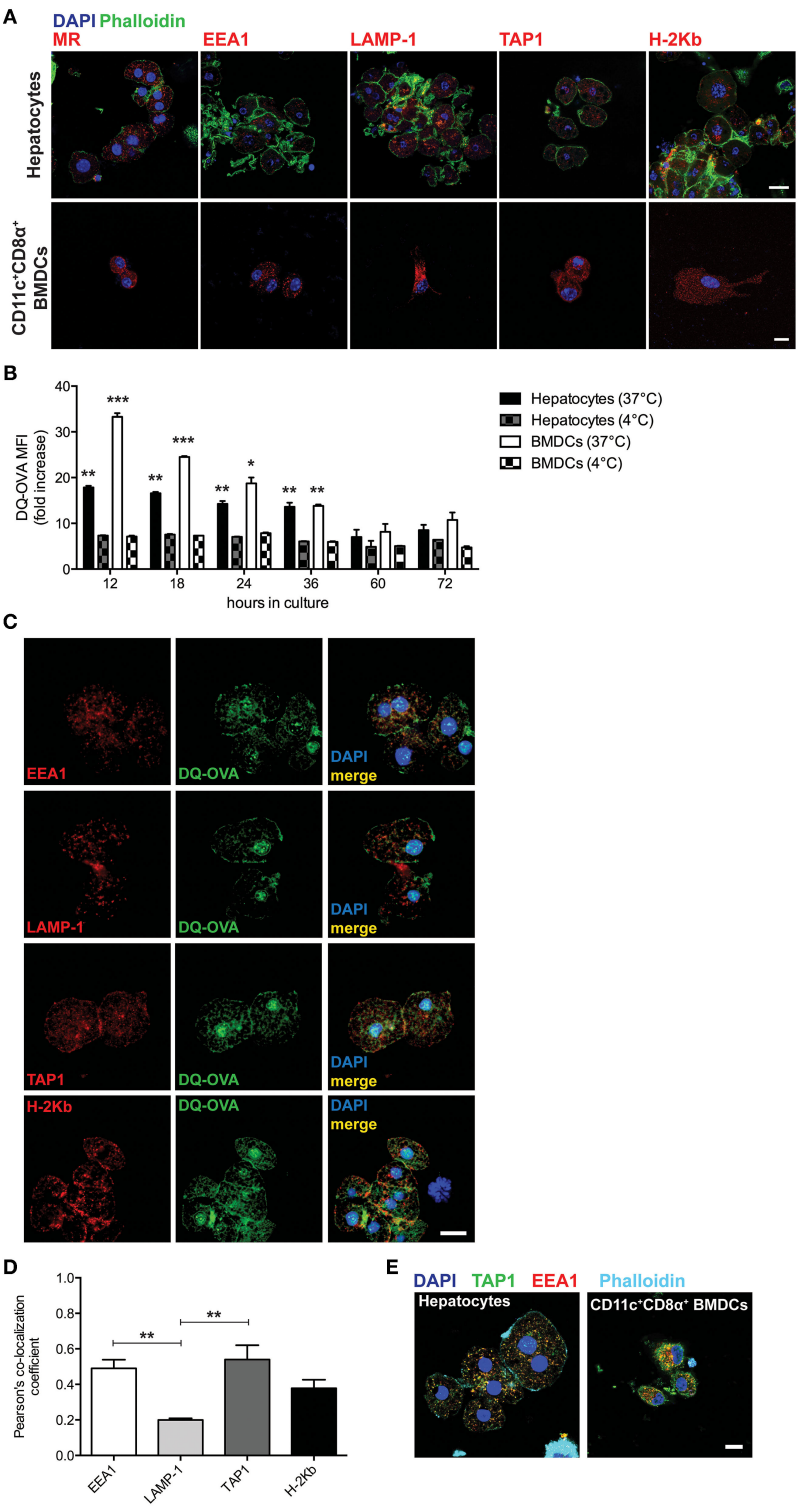
Hepatocytes take up and process extracellular antigens in cross-presentation-competent phagosomes. **(A)** Primary hepatocytes isolated from C57BL/6 mice contain subcellular organelles staining positive for MR, EEA1, LAMP-1, TAP1, and H-2Kb, which are typically associated with antigen cross-presenting functions. Sorted CD11c^+^CD8α^+^ BMDCs from C57BL/6 mice were chosen as a positive control. Scale bar = 10 μm. **(B)** The fluorescent signal originated from the intracellular degradation of DQ-OVA is detected by flow cytometric analysis of primary hepatocytes or BMDCs from C57BL/6 mice cultured for the indicated amount of time in the presence of 20 μg/ml DQ-OVA at 37°C but not at 4°C. **(C)** In primary C57BL/6 hepatocytes, DQ-OVA fluorescent signal is localized in the proximity of or inside EEA1^+^, LAMP-1^+^, TAP1^+^, and H-2Kb^+^ phagosomes. Scale bar = 10 μm. **(D)** Quantification of signal co-localization of DQ-OVA with either EEA1, LAMP-1, TAP1, or H-2Kb as detected by confocal microscopy in **(C)** indicates DQ-OVA degradation is mainly found in EEA1^+^ and TAP1^+^ phagosomes. **(E)** EEA^+^TAP1^+^ phagosomes, typical of professional antigen cross-presenting cells as indicated by staining of CD11c^+^CD8α^+^ BMDCs, are found in the cytoplasm of primary hepatocytes. Scale bar = 10 μm. *P < 0.05, **P < 0.01 and ^***^P < 0.001 [unpaired Student's *t*-test in **(B)** and one-way ANOVA and Bonferroni *post-hoc* test correction in **(D)**. Data in **(A–D)** are representative of 3 independent experiments [mean and s.e.m. in **(B,D)**].

We first sought to characterize primary murine hepatocytes for their expression and distribution of markers associated with cross-presentation-competent phagosomes. Freshly isolated hepatocytes from C57BL/6 mice (Supplementary Figure 1) were stained for mannose receptor 1 (MR), EEA1, TAP1, and H-2Kb and were analyzed by confocal microscopy. We compared primary hepatocytes to sorted CD11c^+^CD8α^+^ bone marrow-derived dendritic cells (BMDCs) (professional antigen cross-presenting cells) and were able to observe that primary hepatocytes also contain cytoplasmic organelles staining positive for such markers (Figure 1A).

To test whether the subcellular compartments described in Figure 1A are used by hepatocytes to process protein antigens found in the extracellular space, we cultured primary hepatocytes in the presence of DQ-Ovalbumin (DQ-OVA) and analyzed the mean fluorescence intensity (MFI) and localization of its fluorescent signal that is the consequence of its degradation into peptides. Hepatocytes actively degrade proteins added to their supernatant, as DQ-OVA becomes fluorescent when cells are incubated at 37°C but not at 4°C (Figure 1B). By confocal microscopy, we could localize the fluorescent signal originating from the degradation of DQ-OVA in the proximity of or inside EEA1^+^, TAP1^+^, and H-2Kb^+^ compartments (Figure 1C). Quantification of co-localizing signals indicated that DQ-OVA degradation is mainly associated with EEA1^+^ and TAP1^+^ compartments (2.45 and 2.7 Pearson's co-localization coefficient fold increase over LAMP-1, respectively) (Figure 1D). This observation prompted us to investigate whether hepatocytes contain phagosomes positive for both EEA1 and TAP, which are considered the hallmark of professional cross-presenting cells, as these phagosomes retain all the functions necessary for cross-presentation (19–24). Interestingly, EEA1^+^TAP1^+^ subcellular compartments are abundantly distributed in the cytoplasm of primary hepatocytes, even though to a lesser extent as compared to sorted CD11c^+^CD8α^+^ BMDCs (Figure 1E). These results are in line with the current models of cross-presentation, according to which cross-presenting cells contain phagosomes (mainly recognized as EEA1^+^) equipped with the complete molecular machinery necessary to retro-translocate antigens to the cytoplasm for degradation into phagosome-associated proteasomes and to transport digested peptides into endosomal MHC-I-containing compartments, where peptides are loaded onto MHC-I complexes prior to their transportation to the cell membrane (25, 27).

**Figure 2:**
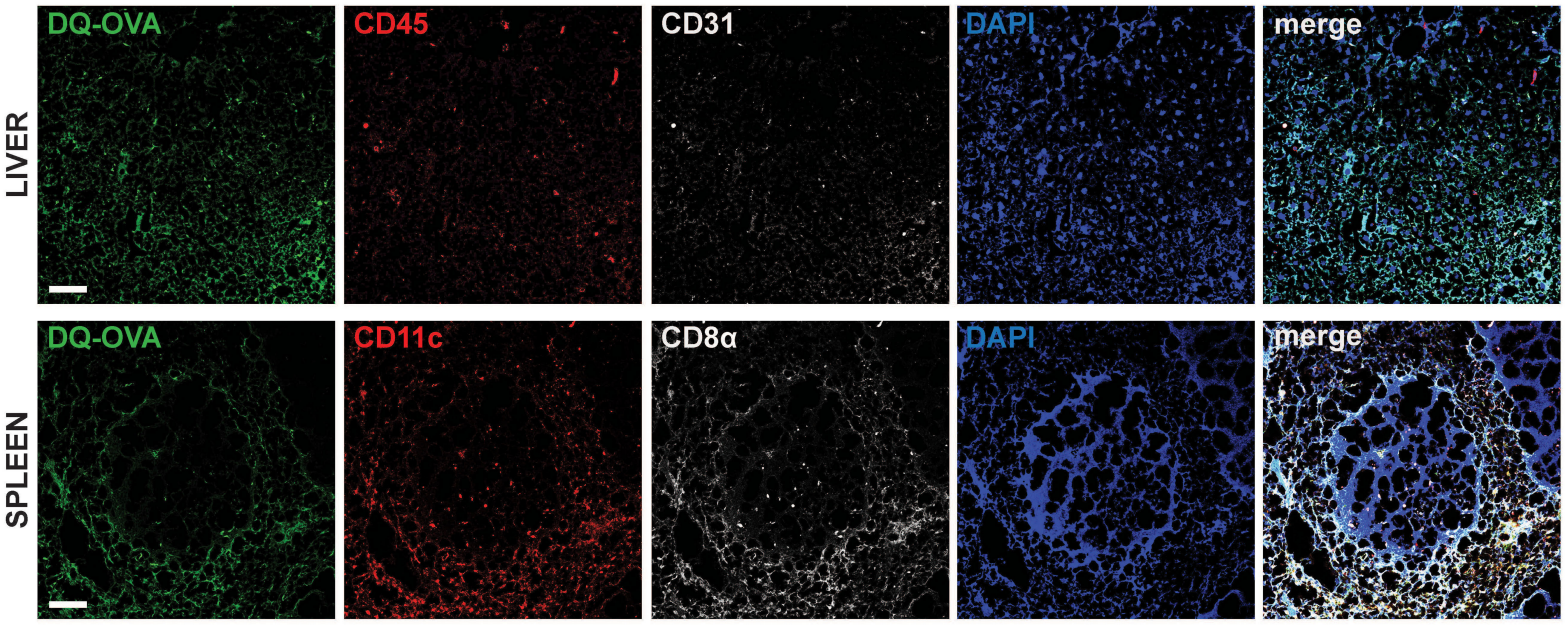
Intravenously administered soluble antigens are processed by liver CD45^−^CD31^−^parenchymal cells and CD11c^+^CD8α^+^ splenocytes. Twelve hours after i.v. administration to C57BL/6 mice, processed DQ-OVA is found within CD45^−^CD31^−^ parenchymal cells of the liver (hepatocytes) (top row) and in CD11c^+^CD8α^+^ cells of the spleen (bottom row). Scale bar = 50 μm. Pictures are representative of 5 different mice.

To confirm *in vivo* uptake and processing of blood-borne extracellular antigens by hepatocytes, we injected DQ-OVA intravenously (i.v.) into C57BL/6 mice and euthanized animals after 12 h to harvest liver and spleen, as they represent the major blood-filtering organs. In the liver, DQ-OVA fluorescence was widely distributed in the parenchyma and mainly localized in hepatocytes, identified as non-hematopoietic (CD45^−^) non-endothelial (CD31^−^) parenchymal cells (Figure 2, top panels). As expected, DQ-OVA processing in the spleen was mostly detected in cross-presenting DCs, identified as CD45^+^CD11c^+^CD8α^+^ cells (Figure 2, bottom panels).

We thus concluded that murine hepatocytes phenotypically resemble antigen cross-presenting DCs, since actively processed extracellular antigens can be mostly detected in association with EEA1^+^, TAP1^+^, and H-2Kb^+^ cytoplasmic compartments. Most importantly, hepatocytes contain EEA1^+^TAP1^+^ phagosomes, which are considered a unique feature of professional cross-presenting cells. Moreover, scavenging and degradation of blood-borne antigens *in vivo* are mainly attributable to hepatocytes in the liver and to cross-presenting CD45^+^CD11c^+^CD8α^+^ DCs in the spleen.

### Polymer With Side-Chain N-Acetylgalactosamine (pGal) Covalently Conjugated to a Protein Antigen Increases the Efficiency of Antigen Cross-Presentation in Primary Hepatocytes

Receptor-mediated endocytosis of extracellular antigens is the first step of the cross-presentation pathway. Antigen chemical modifications enhancing receptor binding have been previously exploited to improve either CD8^+^ T cell immunity or tolerance following antigen cross-presentation (28, 29). We therefore decided to test whether an antigen covalently modified with a water-soluble polymer functionalized with side-chain N-acetylgalactosamine residues (abbreviated pGal, 25), which is recognized by several cross-presentation-related scavenger receptors including the MR (30), the fructose receptor (31) and the liver-specific lectin ASGPR (32), could improve hepatocyte cross-presentation of the model antigen OVA.

**Figure 3:**
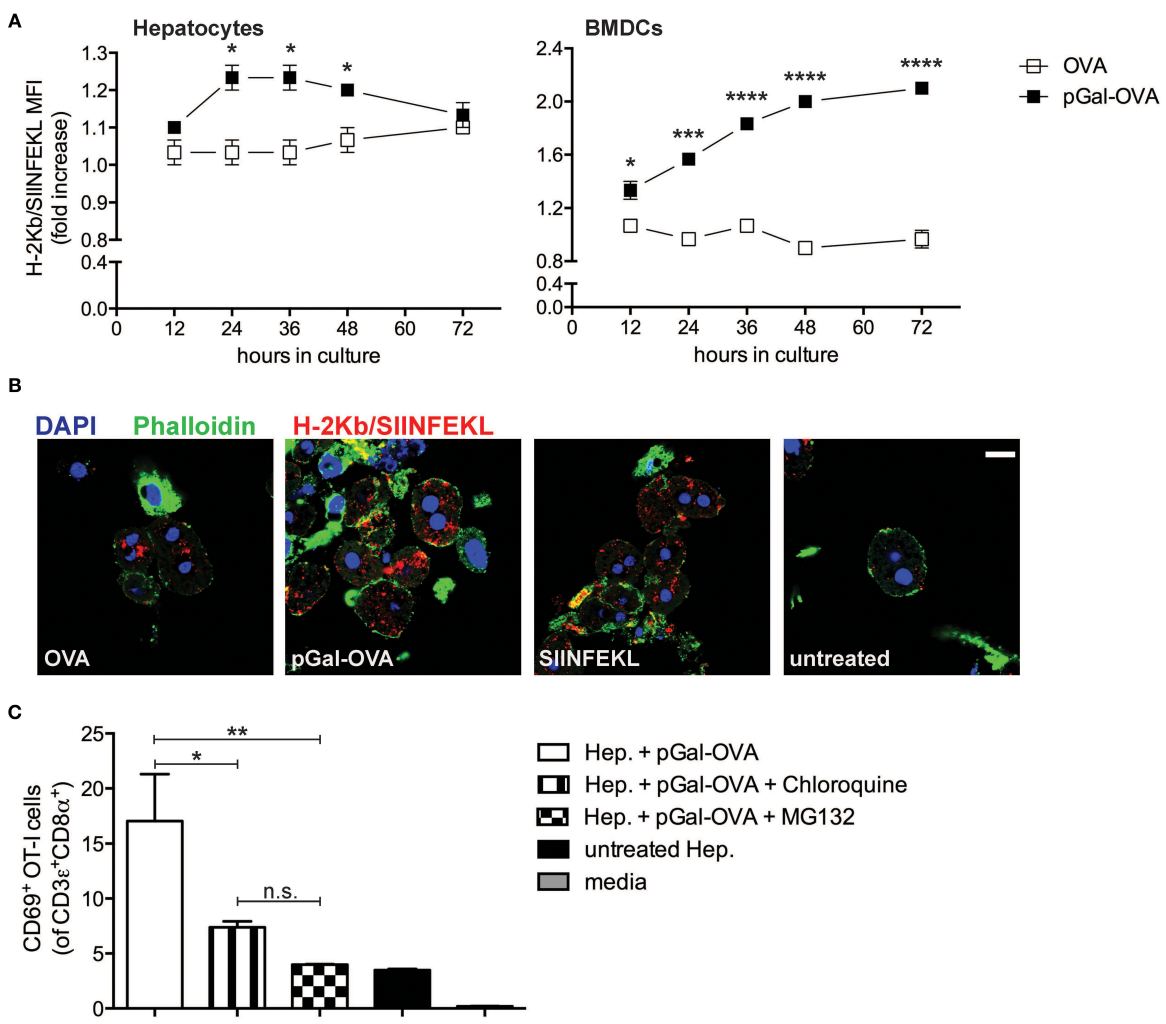
Polymer containing side-chain N-acetylgalactosamine (pGal) conjugate with OVA improves cross-presentation of extracellular OVA. **(A)** Culture of primary hepatocytes (left) or BMDCs (right) from C57BL/6 mice with pGal-OVA (black squares) increases the amount of H-2Kb-bound SIINFEKL detected by flow cytometric analysis as compared to culture with unmodified OVA (white squares). **(B)** H-2Kb/SIINFEKL staining of C57BL/6 primary hepatocytes after 24 h culture in the presence of either 5 μM OVA, 5 μM pGal-OVA, 1 nM OVA_257−264_, i.e., SIINFEKL, or left untreated confirms efficient cross-presentation of pGal-OVA. Scale bar = 10 μm. **(C)** Treatment of primary hepatocytes with either chloroquine or MG132 significantly reduces the cross-presentation of pGal-OVA by primary hepatocytes to H-2Kb/SIINFEKL-specific OT-I cells, as indicated by staining for the early T cell antigen-sensing and activation marker CD69 on the surface of OT-I cells. ^*^P < 0.05, ^**^P < 0.01, ^***^P < 0.001, ^****^P < 0.0001 and n.s., not significant (one-way ANOVA and Bonferroni *post-hoc* test correction). Data are representative of 3 independent experiments [*n* = 3; mean and s.e.m. in **(A,C)**].

We modified OVA with pGal (pGal-OVA) and compared its cross-presentation to that of unmodified OVA in hepatocytes or BMDCs incubated with equimolar doses of the unmodified OVA or pGal-OVA antigen (Figure 3A and Supplementary Figure 2). pGal-OVA resulted in a statistically significant 1.2- and 2.1-fold increase of cross-presentation of the OVA-derived CD8^+^ T cell immunodominant epitope SIINFEKL in primary hepatocytes and BMDCs, respectively, as compared to OVA, as indicated by immunostaining for H-2Kb/SIINFEKL pMHC complexes and flow cytometric analysis (Figure 3A). Confocal microscopy confirmed enhanced SIINFEKL cross-presentation by pGal-OVA-treated hepatocytes as compared to OVA-treated hepatocytes (Figure 3B).

We then asked whether cellular processes described in hematopoietic and non-hematopoietic APCs for cross-presentation, such as endosomal acidification and proteasomal degradation (25–27, 33), were also employed by hepatocytes for pGal-OVA cross-presentation. We therefore treated primary murine hepatocytes with pGal-OVA alone or with pGal-OVA together with either chloroquine (an inhibitor of endosomal acidification) or MG132 (a proteasome inhibitor) and cultured them *in vitro* with OT-I cells, transgenic CD8^+^ T cells specific for H-2Kb/SIINFEKL. After 24 h of co-culture, we analyzed by flow cytometry the expression of CD69 by OT-I cells, as an early indicator of antigen sensing and TCR triggering. Blockade of either endosomal acidification or proteasomal protein degradation in hepatocytes resulted in statistically significant reduction of the frequency of OT-I cells able to experience antigen presentation by hepatocytes, as indicated by CD69 staining. In fact, after treatment of hepatocytes with pGal-OVA and either chloroquine or MG132, 7.38 and 3.98% of the OT-I cells were CD69^+^, respectively, as compared to 17% of CD69^+^ OT-I cells measured when hepatocytes were incubated with pGal-OVA alone (Figure 3C).

These data indicate that pGal-OVA is processed in hepatocytes via the cellular pathway of antigen cross-presentation. Since the pGal modification of a protein antigen, in our case OVA, results in more efficient scavenging and subsequent cross-presentation of the antigen itself, we decided to adopt pGal-OVA as model antigen to characterize the antigen-specific immune response elicited by hepatocyte-dependent cross-presentation *in vivo*.

### Cross-Presentation of OVA by Hepatocytes Results in Antigen-Specific CD8^+^ T Cell Tolerance of Adoptively Transferred OT-I Cells

Scavenger receptors are expressed by a multitude of cells, especially in the liver and in the spleen, including DCs, macrophages, and LSECs. To discriminate the role of hepatocytes in the establishment of cross-tolerance toward extracellular antigens using the pGal-OVA antigen construct, we developed a model of i.v. adoptive transfer of freshly isolated and antigen-experienced hepatocytes. When delivered i.v., CFSE-labeled primary hepatocytes appear to home to the spleen and, to a lesser extent, the liver, and to survive in those sites for at least 1 month after infusion (Supplementary Figure 3).

**Figure 4:**
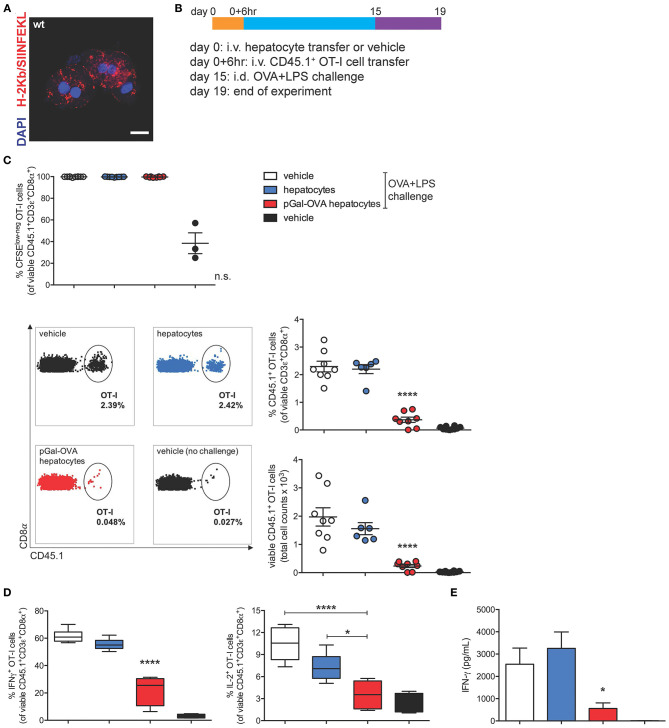
OVA cross-presenting hepatocytes induce CD8^+^ T cell tolerance of adoptively transferred OT-I cells. **(A)** Confocal microscopy of primary hepatocytes from C57BL/6 mice incubated *ex vivo* for 3 h with 12.5 μM pGal-OVA and stained for H-2Kb/SIINFEKL and with DAPI. Scale bar = 10 μm. **(B)** Experimental design. Hepatocytes were exposed to antigen *ex vivo*, prior to intravenous infusion. **(C)** Proliferation (measured as CFSE dilution) (top) and frequency or total cell counts (bottom) of viable CD3ε^+^CD8α^+^CD45.1^+^ OT-I cells were measured by flow cytometry after harvesting from the dLNs of recipient CD45.2^+^ C57BL/6 mice treated as indicated in **(B)**. Numbers in the representative dot plots indicate the frequency of CD45.1^+^ OT-I cells in the population of viable CD3ε^+^CD8α^+^ cells. **(D)** Upon *ex vivo* restimulation with OVA_257−264_ (SIINFEKL), viable CD3ε^+^CD8α^+^CD45.1^+^ OT-I cells harvested from the dLNs of CD45.2^+^ C57BL/6 mice treated as in **(B)** were stained intracellularly for IFN-γ (left) or IL-2 (right) and analyzed by flow cytometry. **(E)** IFN-γ secreted by total dLN cells harvested from treated CD45.2^+^ C57BL/6 mice and restimulated with SIINFEKL was quantified by ELISA. ^*^P < 0.05, ^****^P < 0.0001 and n.s., not significant for comparisons of pGal-OVA hepatocyte-treated group with either vehicle (plus challenge)- or hepatocyte-treated group (one-way ANOVA and Bonferroni *post-hoc* test correction). Data are representative of 2 independent experiments (*n* = 8; mean and s.e.m. in **C–E**).

To study the effects of hepatocyte cross-presentation on antigen-specific T cells *in vivo*, we *ex vivo* incubated pGal-OVA with hepatocytes isolated from C57BL/6 mice. After incubation with pGal-OVA and washing, OVA cross-presenting hepatocytes (Figure 4A) were transferred i.v. into recipient CD45.2^+^ C57BL/6 mice, followed by i.v. administration of CFSE-labeled CD45.1^+^ OT-I cells 6 h later. Two weeks after hepatocyte and OT-I cell transfer, recipient mice were vaccinated with an intradermal (i.d.) dose of OVA and LPS (antigen challenge) into the frontal footpads, and 4 days after challenge mice were euthanized to analyze the phenotype of adoptively transferred OT-I cells retrieved from the spleen and the LNs draining the vaccination site (dLNs) (Figure 4B).

95 to 99.7% of the OT-I cells harvested from mice challenged with OVA/LPS on day 15 responded to vaccination by proliferating, as detected by flow cytometric analysis of CFSE dilution of viable CD45.1^+^CD3^+^CD8^+^ cells in the dLNs and spleen of recipient mice (Figure 4C, top panel, and Supplementary Figure 4A, respectively). Even though no difference was detectable in the proliferative capacity of OT-I cells harvested from vaccinated mice administered on day 0 with either vehicle, untreated hepatocytes or OVA cross-presenting hepatocytes, the frequency of CD45.1^+^ OT-I cells in the population of total viable CD3^+^CD8^+^ lymphocytes was significantly reduced to 0.4% in the dLNs of mice treated with OVA cross-presenting hepatocytes as compared to mice receiving either vehicle (2.3%) or untreated hepatocytes (2.2%) on day 0 (Figure 4C, bottom panels). Reduced frequencies of CD45.1^+^ OT-I cells in mice pre-treated with OVA cross-presenting hepatocytes were paralleled by lower OT-I cell counts (Figure 4C, bottom panels). Significantly reduced frequency and cell counts of CD45.1^+^ OT-I cells were also measured in the population of viable CD3^+^CD8^+^ lymphocytes isolated from the spleen of recipient mice (Supplementary Figure 4A).

The significantly lower frequencies of CD45.1^+^ OT-I cells among total CD8^+^ T lymphocytes prompted us to investigate whether the remaining OT-I cells displayed a dysfunctional phenotype. Administration of pGal-OVA-treated hepatocytes on day 0 significantly affected the capacity of adoptively transferred OT-I cells to acquire effector functions following the vaccination challenge, as indicated by statistically lower frequencies of IFN-γ^+^ and IL-2^+^ viable CD45.1^+^CD3^+^CD8^+^ OT-I cells in the dLNs (22.2 and 3.5%, respectively), as compared to mice administered on day 0 with either vehicle (61.8 and 10.4% of dLN IFN-γ^+^ or IL-2^+^ OT-I cells, respectively) or untreated hepatocytes (55.3 and 7.2% of dLN IFN-γ^+^ or IL-2^+^ OT-I cells, respectively) (Figure 4D). Similar trends were also detected in the spleen of recipient mice (Supplementary Figures 4B,C). Interestingly, when total dLN cells were restimulated *ex vivo* with SIINFEKL, secretion of IFN-γ was significantly reduced to 560 pg/mL in the supernatant of the cells harvested from mice receiving pGal-OVA-treated hepatocytes, as compared to mice receiving either vehicle or untreated hepatocytes on day 0 (2,540 and 3,250 pg/mL, respectively) (Figure 4E).

Collectively, these results indicate that the survival, phenotype, and functionality of antigen-specific CD8^+^ T cells are significantly affected in mice infused with antigen cross-presenting hepatocytes. Lack of expansion of OT-I cells in mice receiving pGal pre-treated hepatocytes followed by vaccination could be ascribed to either deletion and/or functional impairment of the adoptively transferred antigen-specific T cells. Additionally, total dLN cells, and not only OT-I cells, displayed impaired responsiveness to OVA vaccination as indicated by significantly reduced secretion of IFN-γ upon *ex vivo* restimulation with SIINFEKL (Figure 4E). As expected from the lack of MHC-II expression by hepatocytes, no significant immune effect of pGal-OVA-treated hepatocytes could be detected on CD4^+^ OT-II cells adoptively transferred into recipient mice instead of OT-I cells (Supplementary Figure 4D). This result also seems to rule out a prominent role in *in vivo* tolerance induction of other liver APCs that could potentially contaminate the hepatocyte fraction purified from donor livers (Supplementary Figure 1).

I.v. administered antigen-experienced hepatocytes could be phagocytosed and degraded, and their antigens, including OVA-derived epitopes, could be presented by host scavenger cells in the absence of co-stimulation, resulting in tolerance induction. In order to rule this out and confirm the direct role of hepatocyte-dependent cross-presentation in the establishment of CD8^+^ T cell tolerance, we analyzed the development of cross-tolerance after administration of either pGal-OVA-treated TAP1^−/−^ hepatocytes or of β-2 microglobulin (β2m)^−/−^ hepatocytes into wild-type (wt) recipients (Supplementary Figure 5A), following the same experimental design indicated in Figure 4B.

**Figure 5:**
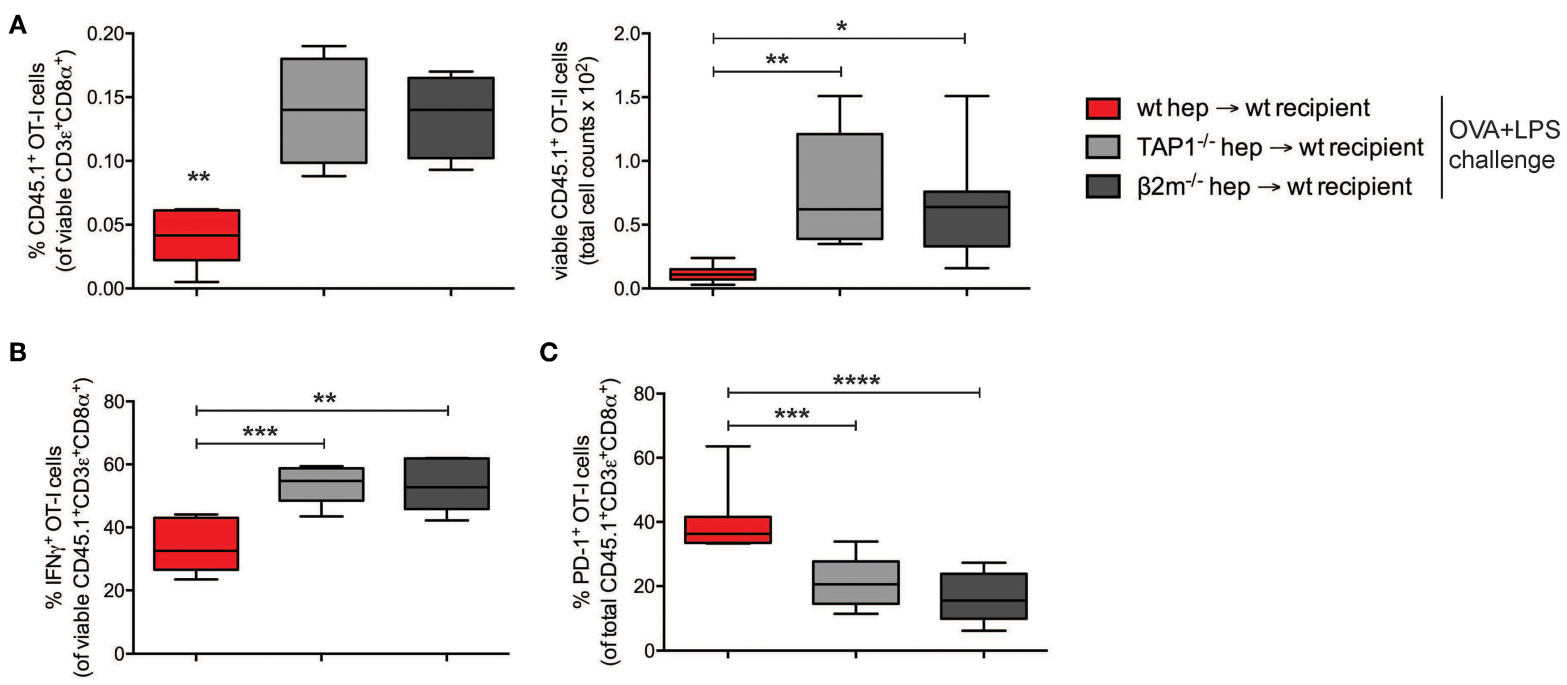
CD8^+^ T cell tolerance is the result of hepatocyte-dependent antigen cross-presentation. **(A)** Frequency (left) and total cell number (right) of viable CD3ε^+^CD8α^+^CD45.1^+^ OT-I cells were analyzed by flow cytometry after harvesting from the dLNs of recipient CD45.2^+^ C57BL/6 mice infused with either wt hepatocytes, TAP1^−/−^ or β2m^−/−^ hepatocytes *ex vivo* incubated with pGal-OVA (12.5 μM) according to the schedule described in Figure 4B. **(B)** Viable CD3ε^+^CD8α^+^CD45.1^+^ OT-I cells harvested from the dLNs of recipient CD45.2^+^ C57BL/6 mice treated as in **(A)** were stained intracellularly for IFN-γ after *ex vivo* restimulation with OVA_257−264_ (SIINFEKL) and analyzed by flow cytometry. **(C)** Viable CD3ε^+^CD8α^+^CD45.1^+^ OT-I cells were stained for PD-1 after harvesting from the dLNs of recipient CD45.2^+^ C57BL/6 mice treated as in **(A)** and analyzed by flow cytometry. ^*^P < 0.05, ^**^P < 0.01, ***P < 0.001, ****P < 0.0001 (unpaired Student's *t*-test). Data are representative of 2 independent experiments (*n* = 8; mean and s.e.m. in **A–C**).

In the dLNs and spleen of mice administered on day 0 with pGal-OVA-experienced TAP1^−/−^ or β2m^−/−^ hepatocytes, the frequency of CD45.1^+^ OT-I cells in the population of viable CD3^+^CD8^+^ lymphocytes was significantly greater than in mice infused with cross-presenting wt hepatocytes (0.14 and 0.13% as opposed to 0.04%, respectively) (Figure 5A). Moreover, the remaining OT-I cells from the dLNs or spleen of mice administered on day 0 with either TAP1^−/−^ or β2m^−/−^ pGal-OVA-treated hepatocytes responded to OVA/LPS challenge more efficiently, as indicated by the increased frequency of IFN-γ-expressing OT-I cells and the decreased frequency of PD-1^+^ OT-I cells detected by flow cytometry in these animals as compared to mice administered with pGal-OVA-treated wt hepatocytes (Figures 5B,C and Supplementary Figures 5C–F).

Altogether, these data confirm the development of cross-tolerance in mice receiving pGal-OVA-treated hepatocytes.

### Antigen Cross-Presenting Hepatocytes Tolerize Endogenous Antigen-Specific CD8^+^ T Lymphocytes and Prevent Acute Rejection of Skin Grafts

**Figure 6:**
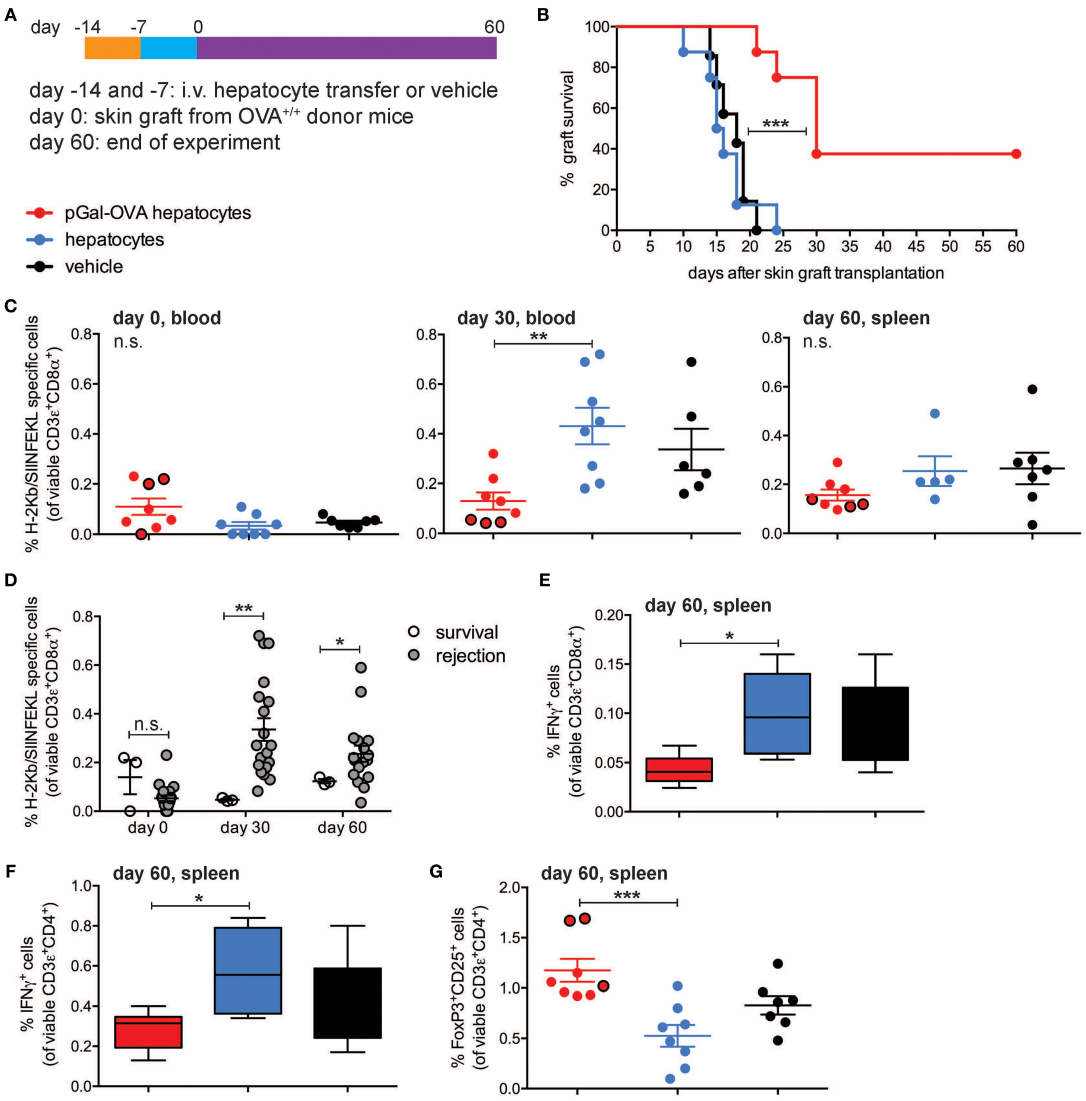
OVA-specific hepatocyte-dependent cross-tolerance prevents acute rejection of skin grafts from OVA+ mice. **(A)** Experimental design, in which hepatocytes are exposed to antigen *ex vivo*, prior to intravenous infusion. **(B)** Survival of the skin from a transgenic mouse expressing transmembrane OVA (OVA^+^ skin) grafted onto wt C57BL/6 mice pre-treated with either pGal-OVA-incubated hepatocytes, untreated hepatocytes or vehicle according to the schedule indicated in **(A)**. **(C)** The frequency of endogenous H-2Kb/SIINFEKL-specific CD3ε^+^CD8α^+^ cells in the blood or spleen of OVA^+^ skin graft recipient mice on day 0 before transplantation (left), day 30 (middle), and day 60 (right) after transplantation was measured by flow cytometry. Dots outlined in black represent mice that retained skin grafts until day 60. **(D)** Frequency of endogenous H-2Kb/SIINFEKL-specific CD3ε^+^CD8α^+^ splenocytes in mice retaining or rejecting the OVA^+^ skin graft as detected by flow cytometry on day 0 (before grafting) and on day 30 in the blood and day 60 in the spleen. **(E)** Viable CD3ε^+^CD8α^+^ splenocytes were analyzed by flow cytometry after harvesting on day 60 from skin-grafted C57BL/6 mice, *ex vivo* restimulation with OVA257-264 (SIINFEKL) and intracellular staining for IFN-γ. **(F)** Viable CD3ε+CD4+ splenocytes were analyzed by flow cytometry after harvesting on day 60 from skin-grafted C57BL/6 mice, *ex vivo* restimulation with OVA_323−339_ (ISQAVHAAHAEINEAGR) and intracellular staining for IFN-γ. **(G)** The frequency of viable FoxP3^+^CD25^+^CD4^+^ T cells was measured by flow cytometric analysis of splenocytes harvested on day 60 from skin-grafted C57BL/6 mice and restimulated with OVA_323−339_. Dots outlined in black represent mice that retained skin grafts until day 60. ^***^P < 0.001 in **(B)** (Log-rank Mantel-Cox test). ^*^P < 0.05, ^**^P < 0.01, ^***^P < 0.001 and n.s., not significant in **(C,E–G)** (one-way ANOVA and Bonferroni *post-hoc* test correction). ^*^P < 0.05, ^**^P < 0.01 and n.s., not significant in **(D)** (Mann-Whitney test). Data are representative of one experiment (*n* = 8; mean and s.e.m. in **C–G**).

To test whether rare endogenous antigen-specific CD8^+^ T lymphocytes could be tolerized by antigen cross-presenting hepatocytes, wt C57BL/6 mice were infused with either pGal-OVA pre-treated hepatocytes, untreated (wt) hepatocytes or vehicle prior to grafting of skin derived from OVA-transgenic (OVA^+^) C57BL/6 mice (Figure 6A). Acute rejection of a transplanted organ typically occurs within the first 3 weeks from grafting as a consequence of host alloreactive T cells recognizing and destroying donor tissues. This is consistent with what we observed in mice administered with either untreated hepatocytes or vehicle prior to grafting OVA^+^ skin, as all of these mice completely rejected the grafted skin by day 24 after transplantation (Figure 6B). On the other hand, acute skin rejection could be delayed and in some cases even prevented in the mice pre-treated with OVA cross-presenting hepatocytes (Figure 6B). Interestingly, 3 out of 8 mice receiving OVA cross-presenting hepatocytes as pre-tolerization treatment retained the OVA^+^ skin grafts until the end of the experimental time on day 60, resulting in a skin graft survival rate of 30% (Figure 6B). The lack of acute skin rejection in the group of mice administered with pGal-OVA-treated hepatocytes was paralleled by lower frequencies of endogenous H-2Kb/SIINFEKL-specific CD8^+^ T lymphocytes as compared to those mice receiving either untreated hepatocytes or vehicle (Figure 6C). In particular, after 30 days from skin transplantation, i.e., at the end of the acute rejection time window, H-2Kb/SIINFEKL-specific CD8^+^ T lymphocytes were 0.13% of viable circulating CD8^+^ T cells in mice that were pre-treated with OVA cross-presenting hepatocytes, as opposed to 0.43 and 0.34% in mice administered with either untreated hepatocytes or vehicle, respectively (Figure 6C, middle). Similarly, at the end of the experimental timeline 60 days after skin transplantation, mice pre-treated with pGal-OVA-incubated hepatocytes still displayed a trend for a lower frequency of H-2Kb/SIINFEKL-specific CD3^+^CD8^+^ splenocytes (0.16%) compared to mice pre-treated with either wt hepatocytes or vehicle (0.25 and 0.27%, respectively), even though differences at this time point did not reach statistical significance (Figure 6C, right). Most importantly, the 3 mice retaining the OVA^+^ skin graft until the end of the experimental time constantly showed significantly lower frequencies of H-2Kb/SIINFEKL-specific CD8^+^ T lymphocytes throughout the entire experimental time (0.047% on day 30 and 0.12% on day 60, in the blood and spleen, respectively) compared to the mice that rejected the OVA^+^ skin graft (0.34% on day 30 and 0.25% on day 60, in the blood and spleen, respectively) (Figure 6D). Reduced frequency of endogenous SIINFEKL-specific CD8^+^ T lymphocytes in mice pre-treated with OVA cross-presenting hepatocytes in this model is reminiscent of the functional impairment observed in adoptively transferred OT-I cells following hepatocyte infusion and vaccination (Figure 4). Moreover, when splenocytes from skin-transplanted mice were restimulated *ex vivo* with SIINFEKL after harvesting on day 60, a significant lower frequency of IFN-γ-expressing CD8^+^ T cells was measured in the samples from mice administered with pGal-OVA-treated hepatocytes (0.043%) as compared to mice receiving either untreated hepatocytes (0.1%) or vehicle (0.09%), indicating anergy as a potential mechanism of tolerance in this model (Figure 6E).

Tolerance of OVA-specific CD8^+^ T lymphocytes can explain the lack of acute skin rejection in the mice pre-treated with OVA cross-presenting hepatocytes, but the lack of chronic rejection in the 3 mice that retained the grafted skin until the end of the experimental time would require additional immune regulatory mechanisms, in particular CD4^+^ T cell tolerance and induction of Treg cells. To investigate whether either of these mechanisms occurred in our experimental setting, total splenocytes harvested from skin-grafted mice on day 60 were restimulated *ex vivo* with the CD4^+^ T cell immunodominant epitope OVA_323−339_ (ISQAVHAAHAEINEAGR), and significantly lower frequencies of IFN-γ-expressing CD4^+^ T lymphocytes were detected in the group of mice receiving OVA cross-presenting hepatocyte pre-treatment (0.28%) as compared to wt hepatocytes (0.58%) or vehicle (0.4%) (Figure 6F). Most importantly, mice administered with pGal-OVA-incubated hepatocytes had significantly higher frequencies of FoxP3^+^CD25^+^CD4^+^ *bona fide* Treg cells (1.18%) as compared to the other treatment groups (0.53 and 0.83% for mice pre-treated with wt hepatocytes or vehicle, respectively), and the frequencies were especially higher in the 3 mice that did not reject the OVA^+^ skin graft (Figure 6G).

These data confirm that hepatocyte-dependent antigen cross-presentation is capable of inducing tolerance of rare endogenous antigen-specific CD8^+^ T cells, avoiding the early post-transplantation phase of antigen-specific cytotoxic T cell-dependent alloreactivity that would otherwise result in acute tissue graft rejection.

## Discussion

Our study described herein highlights the therapeutic feasibility of using hepatocyte-dependent cross-presentation of soluble antigens for the induction of antigen-specific tolerance. Because of its strategic location and microscopic anatomy, the liver has been associated with blood-filtering and immune tolerogenic functions. The hepatic structure is characterized by a complex network of enlarged capillaries, the sinusoids, which are lined by a fenestrated endothelium composed of LSECs and paralleled by plates of hepatocytes (34). LSECs have been so far considered the major contributors to the immunomodulatory functions of the liver, as they are in direct contact with circulating lymphocytes, show efficient antigen scavenging capacity, express both MHC-I and MHC-II, and have low non-inducible levels of co-stimulatory molecules (3–8). On the other hand, hepatocytes only express MHC-I complexes and have been attributed poor antigen scavenging capacity *in vitro*, but efficient CD8^+^ T cell deletion ability *in vivo* upon direct antigen expression and MHC-I presentation in the absence of co-stimulation (10–18).

Given the direct contact that hepatocytes experience with T lymphocytes in the blood, we thus reasoned that they could express and utilize the molecular machinery required for antigen processing and cross-presentation. Cross-presentation of extracellular antigens on MHC-I has been mainly attributed to specialized subsets of hematopoietic cells, in particular lymphoid organ-resident CD8α^+^ DCs (35). Nonetheless, in recent years it has been discovered that subsets of non-hematopoietic cells are also capable of cross-presentation, among which are stromal cells in the LNs and LSECs in the liver (3, 6, 33, 36).

We were able to show that murine primary hepatocytes express high levels of the mannose scavenging receptor 1 (MR) found in other cross-presenting cells and contain abundant cellular compartments positive for markers associated with MHC-I presentation of extracellular antigens, in particular EEA1 and TAP1. Interestingly, hepatocytes were found to contain EEA1^+^TAP1^+^ phagosomes, which are a peculiar characteristic of professional cross-presenting CD11c^+^CD8α^+^ cells (25). We also show both *in vitro* and *in vivo* that hepatocytes actively process extracellular antigens, such as DQ-OVA, especially in association with EEA1^+^ and TAP1^+^ compartments. As expected, the efficiency of antigen processing was less than that in CD11c^+^CD8α^+^ DCs cells, probably due to the higher concentration of cross-presentation-competent phagosomes, mostly EEA1^+^TAP1^+^ compartments, in this DC subset as compared to hepatocytes.

To study how to exploit the cellular machinery for antigen cross-presentation expressed by hepatocytes for tolerogenic purposes, we decided to utilize a derivative of the model antigen OVA chemically modified with a polymer that is functionalized on its side chains with N-acetylgalactosamine (pGal-OVA), which is recognized by several scavenger receptors, including ASGPR on hepatocytes. Our group previously showed that pGal-modified antigens significantly accumulate in liver after *in vivo* administration and can be used to induce antigen-specific immunological tolerance (37). In this study, we showed that pGal-OVA led to improved antigen uptake and consequent enhanced cross-presentation of the OVA-derived immunodominant epitope SIINFEKL by hepatocytes both *in vitro* and *in vivo*.

To specifically characterize the effects of pGal-OVA on hepatocyte-dependent antigen cross-presentation, we adopted an *ex vivo* system where murine hepatocytes are first isolated from the liver of donor mice (38) and incubated with the pGal-OVA antigen, then subsequently washed and infused i.v. into recipient mice (39, 40).

When the phenotype of H-2Kb/SIINFEKL-specific OT-I cells was analyzed after an immunogenic challenge with OVA and LPS in mice receiving hepatocyte transfer, we observed reduced frequencies and numbers of the OT-I cells that had previously experienced cognate antigen presentation by OVA cross-presenting hepatocytes, suggesting an antigen-specific process of T cell tolerance, by either deletion or impaired responsiveness. The remaining hepatocyte-educated OT-I cells showed a phenotype, indicated by low expression of IFN-γ and IL-2 upon *ex vivo* antigen-specific restimulation, further indicating the establishment of tolerance.

Of note, the significant reduction of IFN-γ secretion by total dLN cells harvested from mice receiving OVA cross-presenting hepatocytes as compared to the other treatment groups suggested establishment of hepatocyte-dependent cross-tolerance not only of adoptively transferred OT-I cells but also of endogenous OVA-specific CD8^+^ T lymphocytes. To confirm development of hepatocyte-dependent cross-tolerance by endogenous antigen-specific CD8^+^ T lymphocytes, we infused pGal-OVA-treated hepatocytes prior to grafting OVA^+^ skin into wt recipients and observed prevention of acute rejection of the OVA^+^ skin. The prolonged survival of OVA^+^ grafted skin in mice receiving OVA cross-presenting hepatocytes as compared to mice receiving either vehicle or untreated hepatocytes prior to skin transplantation was associated with reduced frequencies of endogenous H-2Kb/SIINFEKL-specific CD8^+^ T lymphocytes and poor pro-inflammatory cytokine expression in response to *ex vivo* restimulation with SIINFEKL, thus confirming development of cross-tolerance in endogenous OVA-specific CD8^+^ T lymphocytes. Unexpectedly, 3 out of 8 mice receiving OVA cross-presenting hepatocytes prior to skin transplantation retained the OVA^+^ skin until the end of the experimental time 60 days after grafting. Lack of skin graft rejection was associated with higher frequencies of *bona fide* CD4^+^ Treg cells as compared to the mice that instead rejected the transplanted skin. Even though the specific role of CD4^+^ and CD8^+^ T cells in mediating skin graft rejection or survival was not tested in this model, our results suggest that multiple mechanisms, including hepatocyte-dependent cross-tolerogenesis of CD8^+^ T cells, participated in the establishment of immune tolerance toward OVA in those mice that never rejected the OVA^+^ skin grafts. In particular, we hypothesize that the lack of acute skin rejection, made possible by hepatocyte-driven cross-tolerance, created a window of time where graft-derived alloantigens, in our case OVA, could be drained to and presented by host APCs in the absence of danger signals, thus additionally resulting in the development of CD4^+^ T cell tolerance.

Previous publications have shown that the development of peripheral CD4^+^ and CD8^+^ T cell tolerance upon intravenous infusion of antigen-coupled cells depends on the apoptotic phenotype of the transferred cells causing them to be phagocytosed and their antigens to be presented by host APCs in non-inflammatory conditions (41). We thus adoptively transferred pGal-OVA-treated TAP1^−/−^ or β2m^−/−^ hepatocytes into wt recipient mice together with OT-I cells. Impaired antigen cross-presentation by TAP1^−/−^ and β2m^−/−^ hepatocytes resulted in significantly reduced OT-I cell tolerance, confirming the direct role of hepatocyte cross-presentation in tolerogenesis. Moreover, hepatocyte-dependent antigen presentation did not lead to direct CD4^+^ T cell tolerance (with OT-II cells) in our experimental setting, further confirming limited contamination of our hepatocyte preparations by professional APCs. Interestingly, other reports have described the induction of antigen-specific FoxP3^+^ Treg cells upon hepatocyte-specific antigen expression through lentiviral transduction, suggesting that in the case of an antigen directly expressed by hepatocytes, tolerance could result from antigen spreading and MHC-II presentation by other host APCs (16–18).

The establishment of peripheral tolerance is known to be associated with cellular apoptosis. Components derived from apoptotic cells are in fact phagocytosed and subsequently presented by APCs to adaptive immune cells in the absence of pro-inflammatory signals, therefore generating immune tolerance rather than immune activation (42, 43). Apoptotic cells are characterized by peculiar morphological and molecular features, such as activation of neuraminidases. The enzymatic activity of neuraminidases, in turn, is responsible for the removal of terminal sialic acid moieties from cell membrane glycoproteins and glycolipids, leading to exposure of neo-terminal N-acetylgalacosylation on the surface of apoptotic cells and to their subsequent recognition by phagocytotic receptors (44). In this way, pGal-antigen conjugates molecularly mimic the glycated structures exposed on apoptotic debris. By taking advantage of such a chemical tool we could not only confirm previous findings on the ability of hepatocytes to endocytose apoptotic debris (45), but also provide further insights on how to exploit such feature to induce tolerance of CD8^+^ T cells toward an antigen of interest.

In summary, we show here both *in vitro* and *in vivo* that hepatocytes can be used as efficient non-hematopoietic cross-presenting cells for the induction of CD8^+^ T cell tolerance toward soluble antigens. Because of their anatomical location, their abundance and their intense metabolic activities, we propose that hepatocytes are key players in the establishment and maintenance of liver-mediated peripheral tolerance toward exogenous or endogenous extracellular antigens reaching the liver through the bloodstream. Therefore, our data support hepatocytes as interesting candidates for targeted tolerogenic immunotherapies.

## Materials and Methods

### Mice

C57BL/6 mice were obtained from Harlan Laboratories (Gannat, France), C57BL/6 TAP1^−/−^ mice (B6.129S2-*Tap1*^*tm*1*Arp*^/J), C57BL/6 β2m^−/−^ mice (B6.129P2-*B2m*^*tm*1*Unc*^/J), and C57BL/6 OVA^+/+^ mice (C57BL/6-Tg(CAG-OVA)916Jen/J) were purchased from The Jackson Laboratory (Farmington, CT), and CD45.1^+^ OT-I mice were generated by crossing C57BL/6-Tg (TcraTcrb) 1100Mjb (OT-I) mice (The Jackson Laboratories) with CD45.1^+^ C57BL/6-Ly5.1 mice (Charles River, Saint-Germain-Nuelles, France). Eight to twelve week old female mice were used in all animal experiments. Animals were housed in pathogen-free conditions at the animal facility of the Ecole Polytechnique Fédérale de Lausanne and of the University of Chicago. All experiments were performed in accordance with Swiss and US law and with approval from the Cantonal Veterinary Office of Canton de Vaud, Switzerland, and of the Institutional Animal Care and Use Committee (IACUC) of the University of Chicago.

### Cell Isolation and Antigen Loading

Hepatocytes were isolated from the liver of either wt, TAP1^−/−^ or β2m^−/−^ C57BL/6 mice as previously described (38) and cultured on a feeder layer of 3T3 NIH fibroblasts in DMEM medium supplemented with 10% FBS and 100 IU/mL penicillin-streptomycin at 37°C 5% CO_2_. Purity of isolated hepatocyte fractions was confirmed by staining for CD31 and CD45 (BioLegend) followed by flow cytometric analysis (Supplementary Figure 1). BMDCs were generated as previously described (46). To isolate CD11c^+^CD8α^+^ BMDCs, BMDCs were stained with Abs specific for CD11c (BioLegend) and CD8α (Life Technologies) and sorted with a FACSAria cell sorter (BD Biosciences). CD45.1^+^ OT-I cells were purified from the spleen and LNs of CD45.1^+^ OT-I mice by negative selection of CD8α^+^ T cells using the EasySep mouse CD8α T cell isolation kit (Stemcell Technologies) and labeled with CFSE (Life Technologies) following manufacturer's instructions. For *in vitro* analysis of OVA processing, hepatocytes and BMDCs were cultured in complete DMEM or complete RPMI 1640, respectively, supplemented with 20 μg/ml DQ-OVA (Life Technologies). For *in vitro* analysis of SIINFEKL cross-presentation efficiency, hepatocytes, and BMDCs were cultured in 5 μM OVA-, 5 μM pGal-OVA- or 1 nM SIINFEKL-supplemented complete medium for the indicated time and MFI of H2Kb/SIINFEKL surface staining was determined by flow cytometry as detailed below (Supplementary Figure 2 for representative dot plots). Data in Figure 3A show MFI_(treatedsample)_ / MFI_(untreatedsample)._ For *ex vivo* loading with pGal-OVA prior to administration into mice, hepatocytes were isolated from donor mice and incubated for 3 h at 37°C 5% CO_2_ with 12.5 μM pGal-OVA-supplemented complete DMEM (without feeder layer).

### *In vitro* Co-Culture of Hepatocytes and OT-I Cells

Freshly isolated wt C57BL/6 hepatocytes were cultured in 5 μM pGal-OVA-supplemented complete DMEM for 24 h prior to washing and co-culture with 10^5^ OT-I cells for 24 h. For drug inhibitors, chloroquine was used at 100 μM and MG132 at 10 μM and were added 1 h after addition of the antigen for a total of 6 h prior to washing and co-culture with 10^5^ OT-I cells for 24 h. After co-culture, OT-I cells were harvested and stained with Abs specific for the markers CD3ε (eBioscience), CD8α (Life Technologies), and CD69 (BioLegend). Samples were acquired on an LSR II cytometer (BD Biosciences) and data analyzed with FlowJo software (Tree Star).

### Confocal Microscopy and Flow Cytometry of Primary Hepatocytes and BMDCs

Primary hepatocytes were adhered onto 3T3 NIH fibroblast-coated glass coverslips and BMDCs were adhered onto poly-L-lysine-coated glass coverslips. At the end of the experimental procedures, cells were fixed in 4% paraformaldehyde solution, permeabilized in 3% BSA 0.1% saponin PBS and stained with primary Abs specific for MR-1 (AbD Serotec), EEA1 (BioConcept), LAMP-1 (Abcam), TAP1 (Santa Cruz Biotechnology), H-2Kb (BioLegend), or H-2Kb/SIINFEKL (eBioscience) followed by fluorescently labeled secondary Abs (Life Technologies) and fluorochrome-conjugated phalloidin (Life Technologies). Liver, spleen, lungs, and kidneys were harvested after perfusion of euthanized animals with HBSS (Life Technologies). Organs were fixed in 4% paraformaldehyde solution and frozen in OCT (Sakura). Ten micrometer thick sections were sliced and stained with primary Abs specific for PD-L1 or PD-L2 (eBioscience) and fluorescently labeled secondary Abs (Life Technologies) or left unstained. Samples were mounted using ProLong Gold antifade reagent with DAPI (Life Technologies), imaged with a LSM 700 inverted confocal microscope (Zeiss) and data were analyzed with ImageJ software. For flow cytometry, at the end of the experimental procedures, hepatocytes or BMDCs were washed in 2% FBS PBS and acquired on an LSR II cytometer (BD Biosciences) and data analyzed with FlowJo software (Tree Star).

### Hepatocyte Adoptive Transfer

For hepatocyte biodistribution studies, wt C57BL/6 mice were administered i.v. by tail vein injection with 10^6^ CFSE-labeled wt C57BL/6 hepatocytes in 100 μL DMEM. Mice were euthanized after 24 h, 14 day and 1 month to collect liver, spleen, lungs, and kidneys for confocal microscopy. For tolerance studies, on day 0 recipient wt C57BL/6 mice were administered i.v. by tail vein injection with either 10^6^ pGal-OVA-treated or untreated hepatocytes in 100 μL DMEM or with 100 μL DMEM (vehicle) followed by i.v. injection of 3^*^10^5^ CFSE-labeled CD45.1^+^ OT-I cells 6 h later. On day 15, recipient mice were either vaccinated i.d. with 10 μg endo-grade chicken OVA (Hyglos) + 50 ng ultra-pure LPS (InvivoGen) in 50 μL saline divided into the two frontal footpads or left untreated. On day 19, recipient mice were euthanized to collect spleen and brachial and axillary LNs (dLNs). Spleen and dLN single-cell suspensions were either cultured for 6 h at 37°C in the presence of 1 μg/mL SIINFEKL (GenScript) with the addition of 5 μg/mL BFA for the last 3 h of culture for antigen-specific restimulation and intracellular cytokine staining or directly stained for flow cytometry. For flow cytometry analysis, cells were first stained using Live/Dead fixable cell viability reagents (Life Technologies) followed by surface staining with Abs specific for the markers CD45.1 (eBioscience), CD3ε (eBioscience), CD8α (Life Technologies), FasL (BioLegend), TRAIL (BioLegend), KLRG-1 (BioLegend), CD127 (eBioscience), and PD-1 (BioLegend). Staining with biotinylated Annexin V and fluorescently labeled streptavidin (Life Technologies) was performed according to the manufacturer's instructions. For intracellular cytokine staining, cells were fixed in 2% paraformaldehyde solution, permeabilized in 0.5% saponin 2% FBS PBS solution, and incubated with Abs specific for IFN-γ (BioLegend) and IL-2 (eBioscience). Samples were acquired on an LSR II cytometer (BD Biosciences) and data analyzed with FlowJo software (Tree Star). dLN cells were restimulated for 4 days in the presence of 1 μg/mL SIINFEKL for the measurement of IFN-γ by ELISA using the specific Ready-SET-go! ELISA kit from eBioscience.

### Skin Transplantation Studies

On day−14 and−7 recipient wt C57BL/6 mice were administered i.v. by tail vein injection with either 10^6^ pGal-OVA-treated or untreated hepatocytes in 100 μL DMEM or with 100 μL DMEM (vehicle). On day 0, tail skin from donor OVA^+/+^ C57BL/6 mice was grafted onto the back of recipient mice and the survival of the graft was monitored for the following 60 days. Blood was sampled on day 0 (before skin grafting) and on day 30 for flow cytometry analysis of circulating lymphocytes. On day 60, recipient mice were euthanized to collect the spleen. Single-cell suspensions of splenocytes were cultured for 6 h at 37°C in the presence of 1 μg/mL SIINFEKL or ISQAVHAAHAEINEAGR (GenScript) with the addition of 5 μg/mL BFA for the last 3 h of culture for antigen-specific restimulation and intracellular cytokine staining or directly stained for flow cytometry. For flow cytometry analysis, cells were processed as indicated above.

### Statistics

Statistically significant differences between experimental groups were determined by one-way ANOVA followed by either Bonferroni *post-hoc* test correction, unpaired Student's *t*-test, Log-rank Mantel-Cox test or Mann-Whitney test. ^*^P < 0.05, ^**^P < 0.01, ^***^P < 0.001, ^****^P < 0.0001 and n.s. = not significant. Statistical analyses were performed using Prism software (v6.0f, GraphPad Software).

## Data Availability Statement

The original contributions presented in the study are included in the article/Supplementary Material, further inquiries can be directed to the corresponding author/s.

## Ethics Statement

The animal study was reviewed and approved by the Cantonal Veterinary Office of Canton de Vaud, Switzerland, and the Institutional Animal Care and Use Committee (IACUC) of the University of Chicago.

## Author Contributions

MD and JH designed research and wrote the paper. DW synthesized pGal-OVA. MD, DW, and EW performed research. MD analyzed data.

## Conflict of Interest

The EPFL has filed for patent protection on the pGal-antigen delivery platform, and MD, DW, and JH are named as inventors on those patents. Anokion SA and Kanyos Bio, Inc., have licensed those patents, and JH and DW participate in equity in those companies. The remaining author declares that the research was conducted in the absence of any commercial or financial relationships that could be construed as a potential conflict of interest.
