# Supplementary material for: Soluble N-Acetylgalactosamine-Modified Antigens Enhance Hepatocyte-Dependent Antigen Cross-Presentation and Result in Antigen-Specific CD8+ T Cell Tolerance Development
Source: Front Immunol. 2021 Mar 3;12:555095. doi: 10.3389/fimmu.2021.555095 (PMC7965950; doi:10.3389/fimmu.2021.555095)
Supplement: Supplementary file 1 [file Data_Sheet_1.PDF]

Supplementary figures:

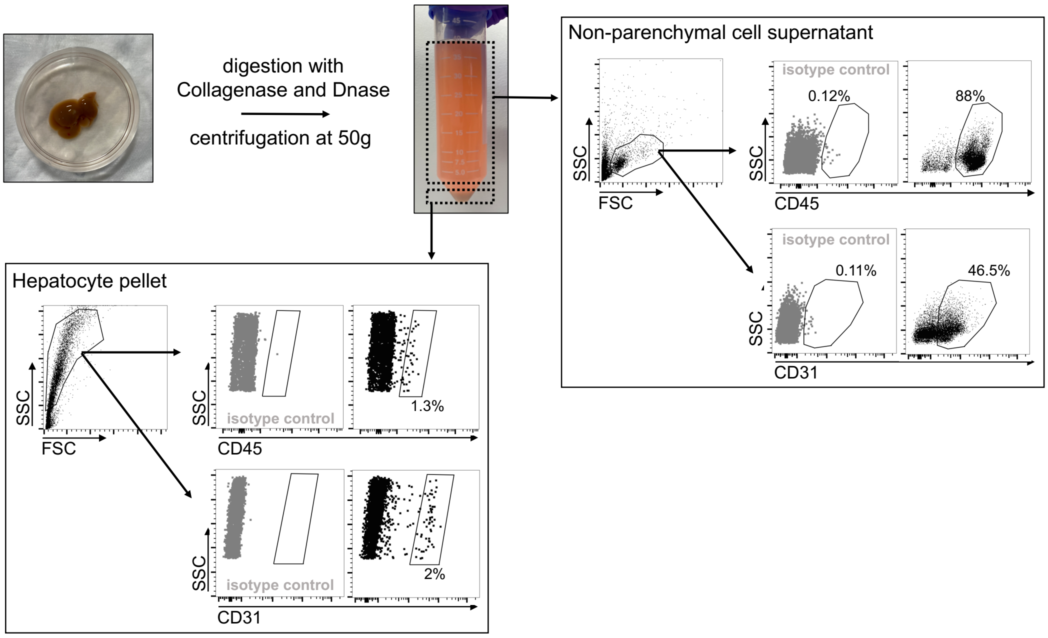

Fig. S1. Isolation of hepatocytes from mouse livers. Flow cytometric analysis of the cell fractions obtained after murine livers were processed as described by Liu W et al. (*45*). Purity of the hepatocyte fraction is confirmed by lack of significant presence of other liver APCs potentially contaminating the hepatocyte pellet. Representative plots, frequencies of gated population are indicated.

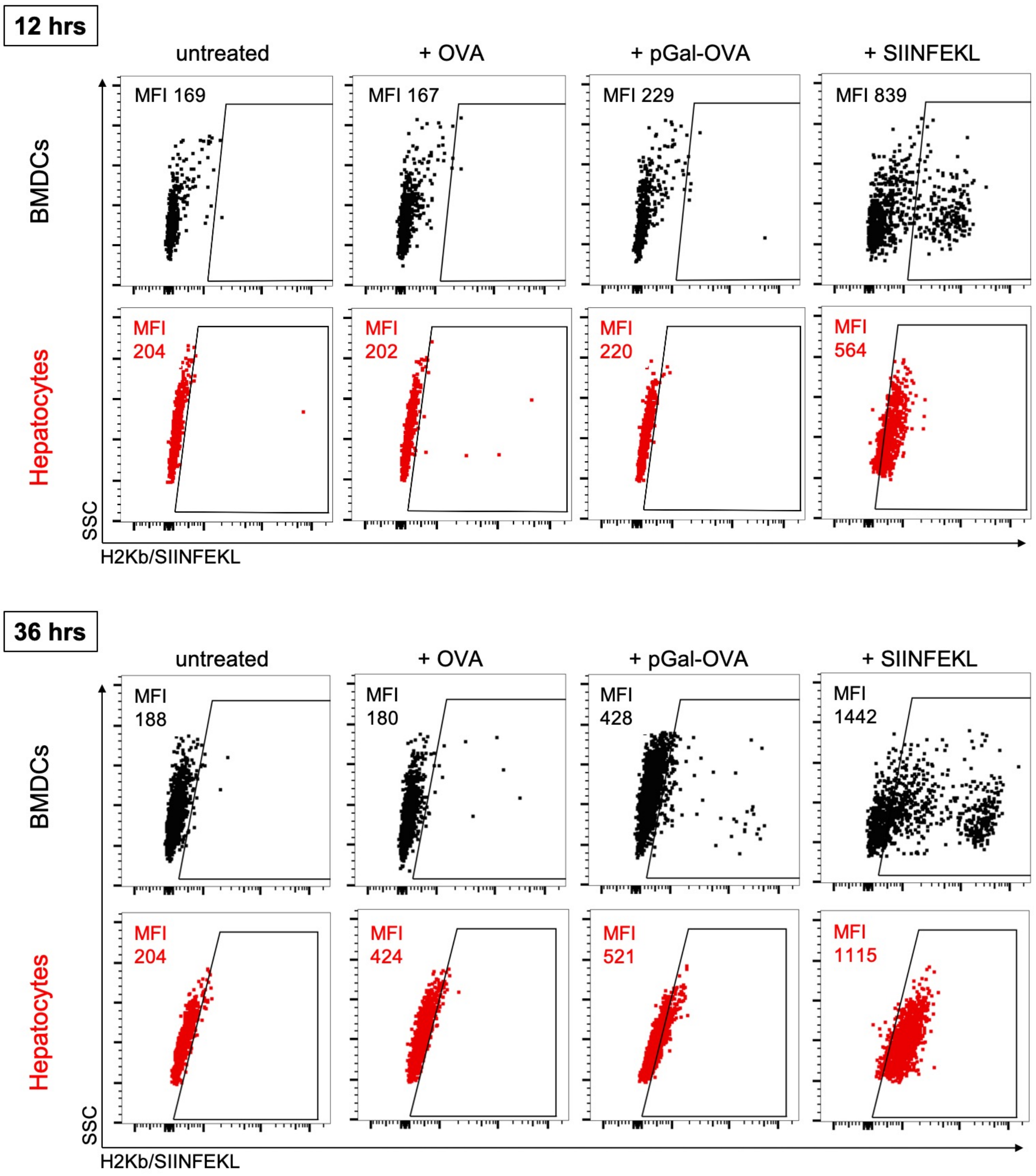

Fig. S2. Comparison of *in vitro* cross-presentation efficiency of OVA and pGal-OVA. Representative dot plots are shown from flow cytometric analysis of BMDCs (black) and primary murine hepatocytes (red) incubated for 12 hrs (top) or 36 hrs (bottom) with either 5 µM OVA, 5 µM pGal-OVA or 1nM SIINFEKL in complete medium or left untreated. After incubation, cells were washed and their surface stained with a H2Kb/SIINFEKL-specific antibody. MFI of total cells is shown.

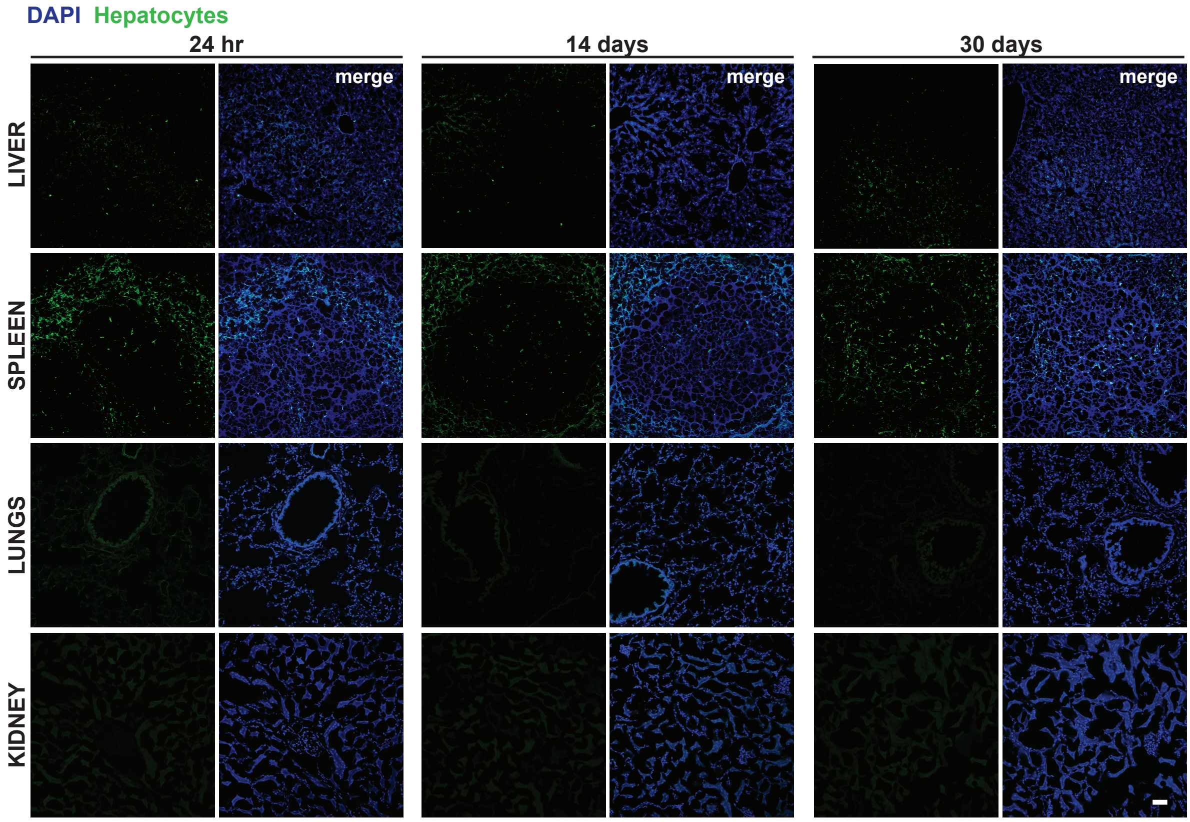

Fig. S3. Primary hepatocytes survive and home to liver and spleen after intravenous transfer. Confocal microscopy of liver, spleen, lung and kidney sections stained with DAPI from C57BL/6 mice administered i.v. with CFSE-labeled C57BL/6 hepatocytes and euthanized at either 24 hr, 14 days or 1 month from injection. Scale bar = 50 μm. Data are representative of 5 different mice.

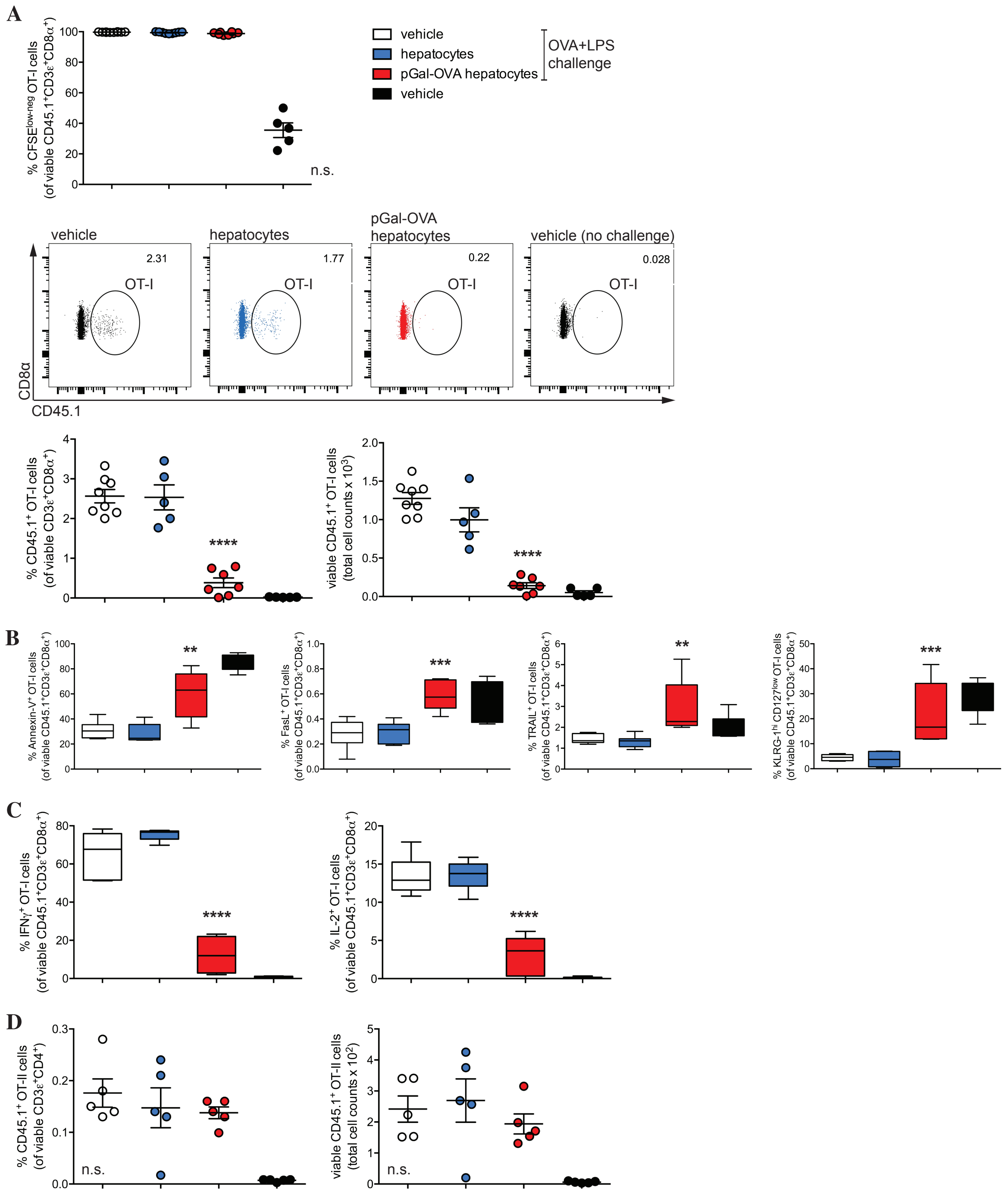

Fig. S4. OVA cross-presenting hepatocytes induce CD8⁺ T cell tolerance of adoptively transferred OT-I cells. (**A**) Proliferation (CFSE dilution) (top), frequency (bottom left) and total number (bottom right) of viable CD3ε⁺CD8α⁺CD45.1⁺ OT-I cells harvested from the spleen of recipient CD45.2⁺ C57BL/6 mice treated as indicated in Fig. 4B were analyzed by flow cytometry. Numbers in the representative dot plots indicate the frequency of CD45.1⁺ OT-I cells in the population of viable CD3ε⁺CD8α⁺ cells. (**B**) Viable CD3ε⁺CD8α⁺CD45.1⁺ OT-I cells harvested from the spleen of CD45.2⁺

C57BL/6 mice treated as in (A) and stained with either Annexin V or for FasL, TRAIL or KLRG-1 and CD127 were analyzed by flow cytometry. (**C**) Viable $CD3\varepsilon^+CD8\alpha^+CD45.1^+$ OT-I cells were stained intracellularly for IFN-γ (left) or IL-2 (right) after harvesting from the spleen of $CD45.2^+$ C57BL/6 mice treated as in (**A**) and *ex vivo* restimulation with $OVA_{257\text{-}264}$ (SIINFEKL). (**D**) The frequency (left) and total cell counts (right) of viable $CD3\varepsilon^+CD4^+CD45.1^+$ OT-II cells harvested from the dLNs of recipient $CD45.2^+$ C57BL/6 mice treated as indicated in Fig. 4B were measured by flow cytometry. $^{**}P < 0.01$, $^{***}P < 0.001$, $^{****}P < 0.0001$ and n.s. = not significant for comparisons of pGal-OVA hepatocyte-treated group with either vehicle (plus challenge)- or hepatocyte-treated group (one-way ANOVA and Bonferroni *post-hoc* test correction). Data are representative of 2 independent experiments ($n = 8$; mean and s.e.m. in A-D).

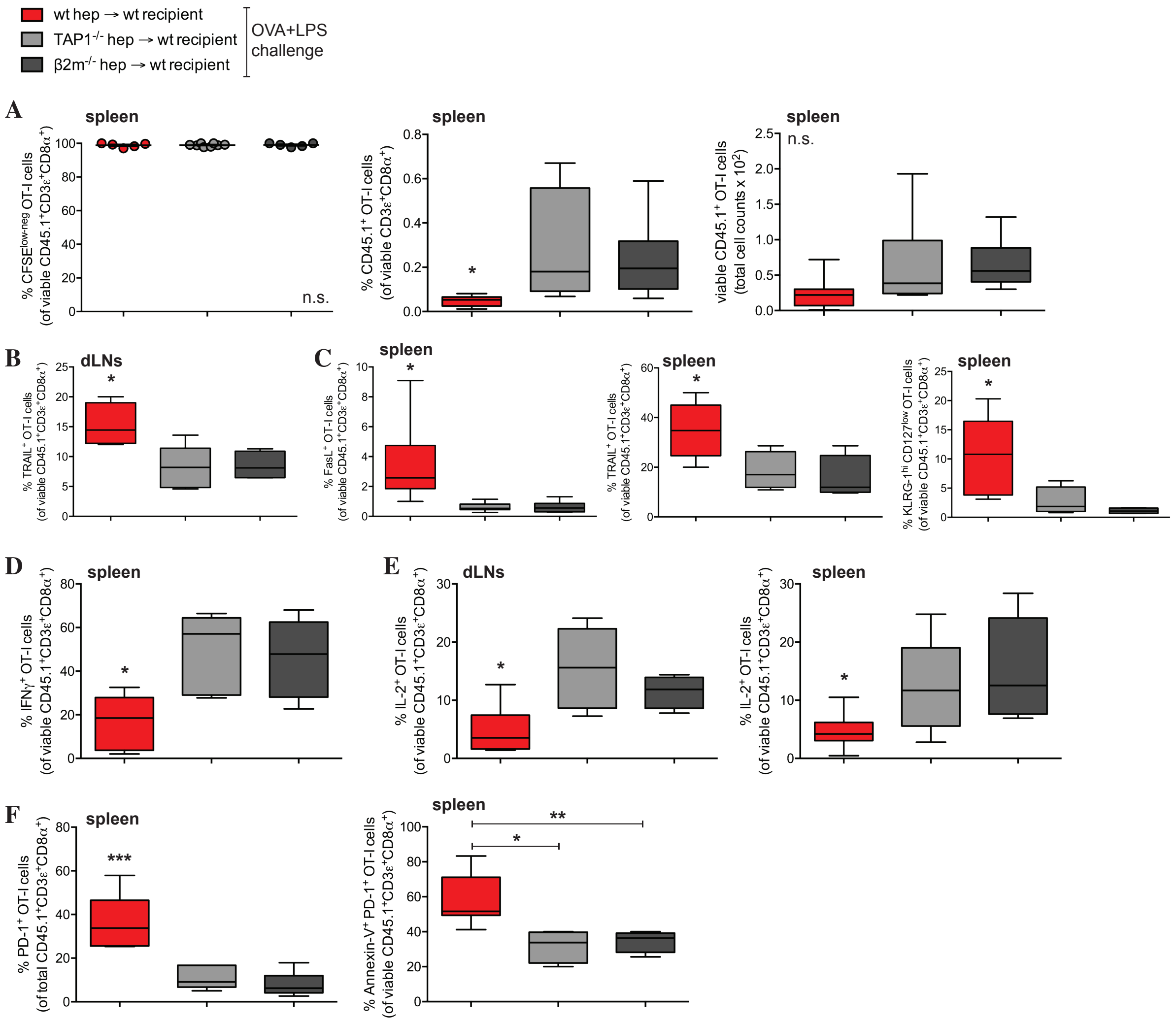

Fig. S5. CD8$^+$ T cell tolerance is the result of hepatocyte-dependent antigen cross-presentation. (**A**) The proliferation (CFSE dilution) (left), frequency (middle) and total number (right) of viable CD3ε$^+$CD8α$^+$CD45.1$^+$ OT-I cells harvested from the spleen of recipient CD45.2$^+$ C57BL/6 mice infused with either wt hepatocytes, TAP1$^{-/-}$ or β2m$^{-/-}$ hepatocytes after *ex vivo* incubation with pGal-OVA (12.5 µM) and treated as described in Fig. 4B were measured by flow cytometry. (**B**) Viable CD3ε$^+$CD8α$^+$CD45.1$^+$ OT-I cells were stained for TRAIL after harvesting from the dLNs of recipient CD45.2$^+$ C57BL/6 mice treated as in (A) and analyzed by flow cytometry. (**C**) Viable CD3ε$^+$CD8α$^+$CD45.1$^+$ OT-I cells were stained for either FasL (left), TRAIL (middle) or KLRG-1 and CD127 (right) after harvesting from the spleen of recipient CD45.2$^+$ C57BL/6 mice treated as in (A) and were analyzed by flow cytometry. (**D**) Viable CD3ε$^+$CD8α$^+$CD45.1$^+$ OT-I cells harvested from the

spleen of recipient CD45.2^{+} C57BL/6 mice treated as in (A) were stained intracellularly for IFN-γ after *ex vivo* restimulation with $OVA_{257\text{-}264}$ (SIINFEKL) and analyzed by flow cytometry. (**E**) Viable CD3ε^{+}CD8α^{+}CD45.1^{+} OT-I cells harvested from the dLNs (right) or spleen (left) of recipient CD45.2^{+} C57BL/6 mice treated as in (A) were stained intracellularly for IL-2 after *ex vivo* restimulation with SIINFEKL. (**F**) Viable CD3ε^{+}CD8α^{+}CD45.1^{+} OT-I cells were stained for PD-1 and with Annexin V and analyzed by flow cytometry after harvesting from the spleen of recipient CD45.2^{+} C57BL/6 mice treated as in (A). The frequencies of PD-1^{+} OT-I cells (left) and of Annexin V^{+}PD-1^{+} OT-I cells (right) are indicated. $^{*}P < 0.05$, $^{**}P < 0.01$ and n.s. = not significant (unpaired t-test). Data are representative of 2 independent experiments ($n = 8$; mean and s.e.m. in A-F).

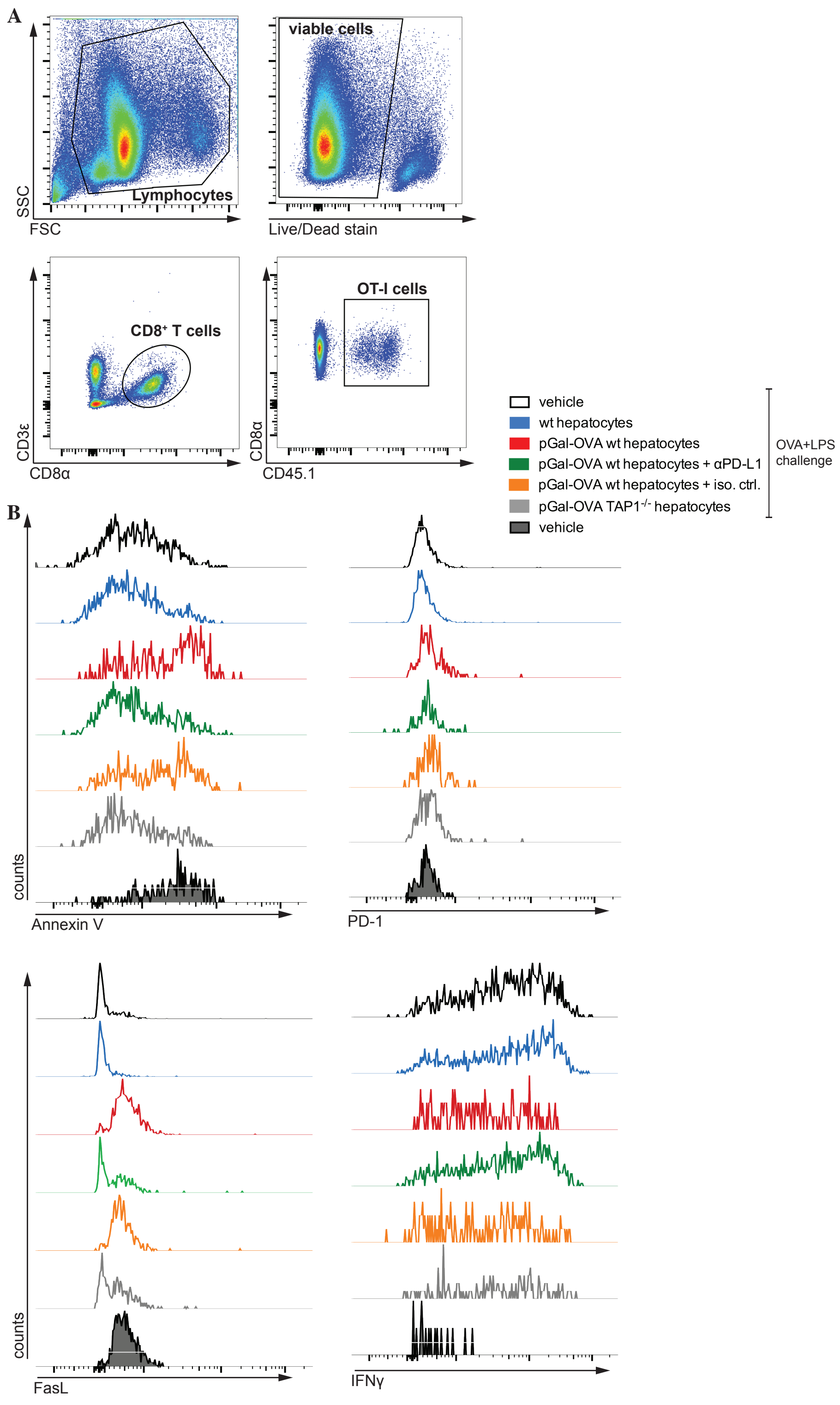

Fig. S6. Flow cytometry gating and examples. (**A**) Gating strategy utilized to identify CD45.1^{+} OT-I cells in CD45.2^{+} C57BL/6 mice in Fig. 5-7 and in Fig. S1-3. Briefly, lymphocytes were gated according to SSC and FSC from total spleen or dLN cells, followed by identification of viable cells, CD3ε^{+}CD8α^{+} T cells and CD45.1^{+} OT-I cells. (**B**) Representative histograms of viable CD3ε^{+}CD8α^{+}CD45.1^{+} OT-I

cell counts positive for either Annexin V (top left), PD-1 (top right), FasL (bottom) or IFN-γ (bottom right) purified from the spleen of CD45.2$^+$ C57BL/6 mice receiving either one of the treatments indicated in the legend following the experimental schedule described in Fig. 5B.
